# Comparison of Prediction Between TIMI (Thrombolysis in Myocardial Infarction) Risk Score and Modified TIMI Risk Score in Discharged Patients From Emergency Department With Atypical Chest Pain

**DOI:** 10.5812/ircmj.13938

**Published:** 2014-02-05

**Authors:** Mohsen Abbasnezhad, Hassan Soleimanpour, Mohamadreza Sasaie, Samad EJ Golzari, Saeid Safari, Maryam Soleimanpour, Robab Mehdizadeh Esfanjani

**Affiliations:** 1 Department of Cardiology, Tabriz University of Medical Sciences, Tabriz, IR Iran; 2 Cardiovascular Research Center, Tabriz University of Medical Sciences, Tabriz, IR Iran; 3 Students’ Research Committee, Tabriz University of Medical Sciences, Tabriz, IR Iran; 4 Medical Philosophy and History Research Center, Tabriz University of Medical Sciences, Tabriz, IR Iran; 5 Department of Anesthesiology and Critical Care, Iran University of Medical Sciences, Tehran, IR Iran; 6 Gastroenterology Research Center, Tabriz University of Medical Sciences, Tabriz, IR Iran; 7 Neurosciences Research Center, Tabriz University of Medical Sciences, Tabriz, IR Iran

**Keywords:** Thrombolysis, Myocardial Infarction, Emergency Department, Atypical Chest Pain

## Abstract

**Background:**

Chest pain is one of the most common causes of the admission to the emergency departments. It, however, can be due to numerous diseases some of which are life threatening.

**Objectives:**

In the current study, we evaluated the prognostic value of TIMI (Thrombolysis in Myocardial Infarction) and Modified TIMI risk scores to stratify the risk for patients with atypical chest pain being discharged from the emergency department.

**Patients and Methods:**

In a prospective-analytic study, we collected data from 1020 patients with atypical chest pain enrolled to the study. All eligible patients were visited by the emergency medicine residents who were trained for this study. Based on the criteria in both systems, the emergency medicine attending decided on either discharging or hospitalizing patients. Patients were allocated into 2 equal groups randomly. In order to predict the opposing accidents in 30 days (coronary revascularization, myocardial infarction, and all-cause death) TIMI risk scores and Modified TIMI risk scores were assessed based on TIMI risk score (0 or 1) and Modified TIMI risk score (0 or 1).

**Results:**

No significant difference could be observed between both groups regarding demographic characteristics, ejection fraction, left ventricle hypertrophy, TRS criteria, risk factors and the history of coronary artery stenosis. None of the atypical chest pain patients discharged based on TIMI and modified TIMI risk scores experienced any adverse events.

**Conclusions:**

The results obtained from this study support the idea that the TIMI and modified TIMI risk scores might be valuable tools that could be used to stratify the risk of patients with atypical chest pain in the emergency department.

## 1. Background

Chest pain is one of the most common reasons of the patients’ referral to the emergency departments (EDs). It, however, can be due to numerous diseases some of which are life threatening. Cardiovascular diseases, aortic dissection, pulmonary emboli, pneumothorax, and pericarditis are some of the fatal diagnoses associated with chest pain. Acute coronary syndrome (ACS), as one of the most important causes of chest pain with a high associated mortality rate, is of great importance and should be diagnosed as early as possible (1). Approach to the chest pain, either typical or atypical, consists of a primary rapid evaluation of differentiating its being typical or a typical. Atypical chest pain cannot rule out MI especially in the female or diabetic patients; therefore, numerous studies have been performed on the more appropriate diagnosis of ACS emergency setting (2).

In other words, atypical chest pain is a common clinical problem which the physicians working in the internal medicine, emergency medicine, and cardiology departments are faced with. Atypical chest pain is mostly seen in the young female patients which is a rarely of the coronary arteries origin. However, whenever of a cardiac origin, it is associated with an additional 7% mortality rate compared with male patients at the same age. Most of these patients undergo angiography to rule out the chance of the coronary artery disease (3). Therefore, one of the major problems is to determine if the existing chest pain is related to ACS or not. A definite criterion is essential to assist us in deciding on if the patient requires hospitalization for further evaluations (4-6).

TIMI Risk Score (TRS) which is used to determine the risk for patients with ACS and is mostly used for patients having chest pain syndrome with unstable angina or myocardial infarction without ST segment elevation (NSTEMI). The scoring uses seven major criteria, each measured as one score, as following (7-11):

1. Age older than 65 years
2. Coronary artery stenosis of more than 50%
3. More than two cardiac disease risk factors
4. History of taking aspirin in the previous seven days
5. Incidence of more than one spells of chest pain within the previous 24 hours
6. ECG changes rather than STEMI
7. Elevated serum levels of cardiac biomarkers

Score Interpretation for TIMI scoring system would be as featured as % risk at 14 days of all-cause mortality, new or recurrent MI, or severe recurrent ischemia requiring urgent revascularization:

Score of 0-1 = 4.7 % risk
Score of 2 = 8.3 % risk
Score of 3 = 13.2 % risk
Score of 4 = 19.9 % risk
Score of 5 = 26.2 % risk
Score of 6-7 = at least 40.9 % risk

The criteria used in Modified TIMI Risk Score (MTRS) are comparable to that of TRS; while in Modified TIMI, the scoring stays between 0-10 and a score of 5, if positive (with a total maximum score of five), reflects cardiac biomarker changes or ECG (6).

## 2. Objectives

In the current study, we evaluated the risk of the patients with atypical chest pain using previously-introduced risk scores of TIMI and Modified TIMI.

## 3. Patients and Methods

In a prospective-analytic study carried out in the ED of Shahid Madani hospital, Tabriz, IR Iran from 2011-2012, patients over 25 years old and with atypical chest pain were included. The Exclusion criteria were as following: age younger than 25 years old, diagnosed STEMI, pregnancy, hospitalization due to other reasons (chronic renal failure requiring dialysis, myocardial contusion, pregnancy), and cases for which follow-up was not practical. This study was approved by the Ethics Committee of “Tabriz University of Medical Sciences” and registered under the Code Number of 8966. After obtaining written informed consents from all patients, ECGs were taken and cardiac Troponin-I enzyme (cTnI) were checked on admission and 12 hours post-symptoms onset. To evaluate cTnI, cTnI ELISA kit (DiaPlis, USA), and to evaluate EKGs ECG monitors (Heart Screen 60-IKO, Innomed, Hungary) were used. All equipment was calibrated prior to administration. Later, patients were allocated into two equal subgroups of I and II randomly by block randomization. Patients in Groups I and II were assessed by TRS and modified TRS, respectively and those with low risk (0-1) were discharged.

**Figure 1.**
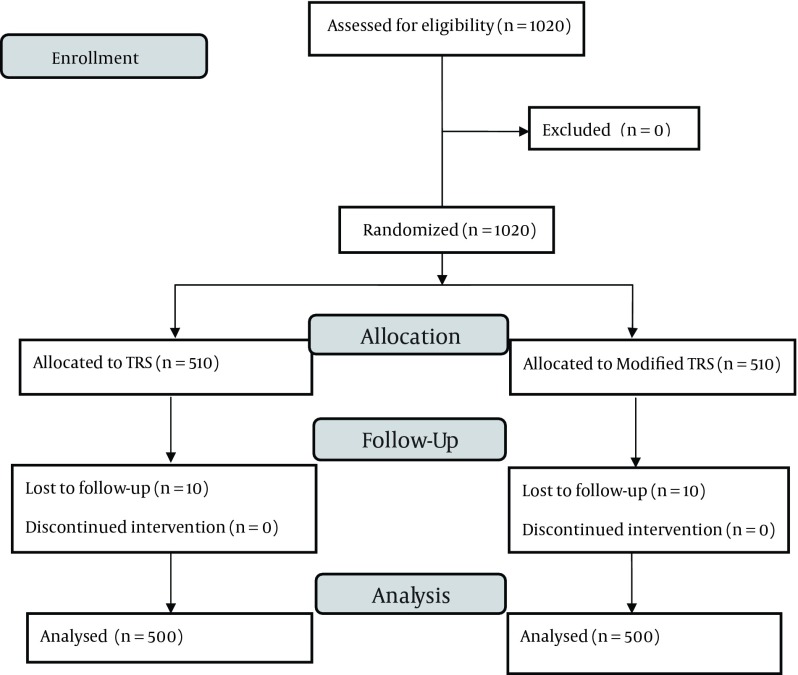
Flow Diagram of Study

All eligible patients were visited by the emergency medicine residents who were trained for this study. Hospitalization or discharge of patients were decided on by the emergency medicine attending (a same attending) based on the two systems. Follow-up via telephone for any probable unwanted complications such as MI, revascularization or death consisted of a one-month period. The data were analyzed using descriptive and deductive-statistical approaches by SPSS ver. 15 software. Variation between qualitative variables and the relation between qualitative and quantitative variables were study using Chi-square and T-test approaches, respectively. The normality of variables was checked by Kolmogorov – Smirnov Z test. Man-Whitney test was used whenever required and P ≤ 0.05 was considered statistically significant. A flow diagram of our study is showed in (Figure 1). 

## 4. Results

**Figure 2.**
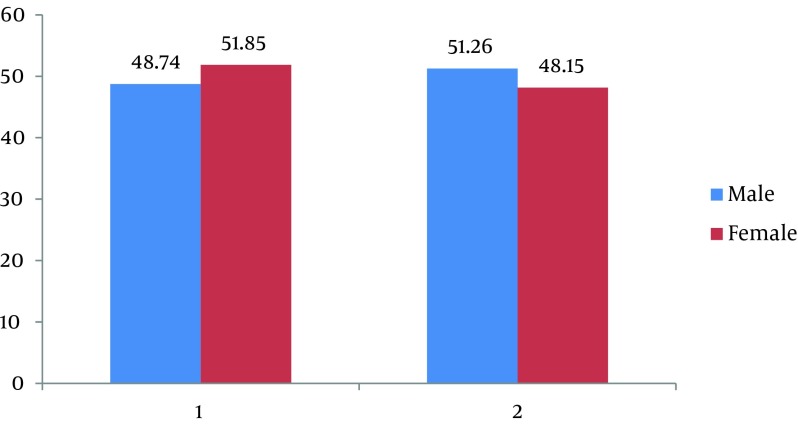
Female to Male Ratio in Both Groups; no Significant Difference Was Observed (P = 0.766)

Within the one-year period of the study, 1020 patients with atypical chest pain were allocated to the study from which 20 people were excluded due to unavailability of follow-up for adverse events (MI, revascularization, death) and finally 1000 people (500 people for each group) including 595 (59.5 %) males and 405 (40.5 %) females were studied. The mean age of the studied patients was 47.72 ± 13.59 years (the youngest and oldest patients were 25 and 91 years old, respectively). Demographic characteristics, ejection fraction, and left ventricle hypertrophy were of no significant difference in both groups (Table 1, Figure 2). 

**Table 1.** Demographic Characteristics and Echocardiographic Findings in Two Groups

	Group I	Group II	P value
**Age Mean ± SD, y**	47.58 ± 56.13	47.84 ± 13.66	0.761
**Ejection Fraction, Mean ± SD**	57.24 ± 4.72	57.25 ± 5.24	0.983

None of the atypical chest pain patients discharged based on TIMI and modified TIMI risk scores experienced any adverse events. No statistically significant difference was observed between both groups regarding TRS criteria, risk factors and the history of coronary artery stenosis; however, a significant difference was detected regarding the number of patients using aspirin (Tables 2 -4). 

**Table 2.** Comparison of the TIMI in Both Groups

TIMI Criteria	Group I, No. (%)	Group II, No. (%)	P value
**More than 3 risk factors**	16 (3.2)	13 (2.6)	0.572
**Age over 65 years old**	51 (10)	39 (7.8)	0.185
**Stenosis history**	9 (1.8)	15 (3)	0.215
**ST segment changes **	8 (1.6)	0	NA
**More than two chest pain reports within previous 24 hours**	11 (2.2)	17 (3.4)	0.250
**Aspirin Consumption**	27 (5.4)	64 (12.8)	< 0.0001
**Increased cardiac enzymes**	0	0	NA

**Table 3.** Comparison of the Risk Factors Between Two Groups

Risk Factors	Group I, No. (%)	Group II, No. (%)	P value
**Hypertension**	165 (33)	148 (29.6)	0.246
**Diabet mellitus**	41 (8.2)	34 (6.8)	0.401
**Cigarette smokers**	97 (19.4)	102 (20.4)	0.692
**Familial history**	27 (5.4)	21 (4.2)	0.375
**Hyperlipidemia**	79 (15.8)	74 (14.8)	0.661

**Table 4.** History of Coronary Artery Stenosis

History of Coronary artery stenosis	Group I, No. (%)	Group II, No. (%)	P value
**Cardiac catheterization**	3 (6)	3 (6)	NA
**Previous angioplasty**	10 (2.0)	4 (8)	0.106
**Coronary artery bypass graft**	2 (4)	10 (2)	0.020

## 5. Discussion

From all patients with atypical chest pain referring to the ED, almost 7% have been reported to have ECGs with the signs of ischemia or MI and only do 6-10% of the patients have primary positive cardiac enzymes. Rest of the patients, despite not fulfilling the required diagnostic criteria, could also have ACS demanding further tests for confirming or ruling out ACS (5). TIMI Risk Score has been reported to be of high success rate in many studies. In a study carried out by Chase et al. it was suggested that the higher the TRS, the more increased the probability of one-month adverse events (death, acute myocardial infarction, and coronary revascularization) (7). In a recent study carried out by Kelly, patients having chest pain with normal ECG, TRS of zero and normal cardiac enzyme levels have been reported to be highly improbable to experience adverse events (8). 

Although TRS enables us to classify the unselected ED chest pain population and achieve solid decisions, it is prone to missing 2-5% of the patients with myocardial infarction (4). In another study by Almagro et al. TRS was shown to be an appropriate predicted tool either in long-term or short-term in patients with atypical chest pain referring to the EDs (12). On the other hand, MTRS has been introduced as a superior scoring system of the risk evaluation in patients with undifferentiated chest pain referring to the EDs (6, 13). Unlike other studies, our study revealed that both TRS and MTRS have the equal predictive values in patients with atypical chest pain referring to the ED and none is superior to the other. Adverse complications could occur both in TRS and MTRS systems requiring precise clinical judgment and multi-element follow up for all patients.

### 5.1. Limitations

One of the limitations of the current study was its being single center studying only Iranian patients. In addition, we were not able to follow almost 20 patients. Another factor which may have posed selection bias leading to misclassification is the fact that our trained researchers were present in the emergency department for only 12 hours a day and seven days a week.

In conclusion Both TRS and MTRS could be used for risk prediction in patients discharged with atypical chest pain from the EDs. However, further future multi-center studies with higher sample volumes are recommended to approve the result obtained from the current study.
